# Prevalence and pregnant women’s knowledge of maternal obesity and excessive gestational weight gain among women attending antenatal care in Fako Division, Cameroon

**DOI:** 10.11604/pamj.2023.44.2.36592

**Published:** 2023-01-03

**Authors:** Ebiambu Ondoh Agwara, Nicholas Tendongfor, Promise Tamunoipiriala Jaja, Anna Maria Choy, Thomas Obinchemti Egbe

**Affiliations:** 1 Faculty of Health Sciences, University of Buea, Buea, Cameroon,; 2 Department of Neurological Surgery, University College Hospital, Ibadan, Nigeria,; 3 Division of Molecular and Clinical Medicine, University of Dundee, Dundee, UK,; 4 Obstetrics and Gynaecology Service, Douala General Hospital, Douala, Cameroon

**Keywords:** Obesity, gestational weight gain, knowledge, pregnant women, antenatal care, Cameroon

## Abstract

**Introduction:**

obesity poses significant public health concerns, being a risk factor for most non-communicable diseases and future cardiovascular diseases. Maternal obesity could be associated with adverse maternal-foetal outcomes, and there is a scarcity of data regarding obesity in pregnancy in our setting. Our objective was to determine the prevalence and knowledge of obesity and excessive Gestational Weight Gain (GWG) among pregnant women attending ANC in the Fako Division.

**Methods:**

we conducted a hospital-based cross-sectional study from January 28 to May 29, 2020, in the Limbe District Hospital (LDH) and Buea Road Integrated Health Centre (BRIHC). We collected data on socio-demographic prevalence, including knowledge of obesity and excessive GWG among pregnant women. Data was analysed using IBM SPSS version 26.

**Results:**

out of the 317 participants included, 58.9% (n=185) were aged 20-29 years, 36% (n=116) unemployed. The mean gestational age was 28.82 ± 7.75 weeks and 33.1% (n=105) were nulliparous. The prevalence of obesity in pregnancy and excessive GWG were 42.3% (n=134) and 41.6% (n=132) respectively. Respondents who consumed alcohol were more likely to be obese (aOR: 2.11, 95% CI 1.19-3.71; p; = 0.01). Those aged <20 (aOR: 0.064, 95% CI 0.007-0.57; p= 0.014) and 20-29 years (aOR: 0.297, 95% CI 0.16-0.56; p<0.001) were less likely to be obese than those 30-39 years. 46.1% (n=147) had poor knowledge of the complications of obesity in pregnancy, while 77.3% (n=245) had moderate knowledge of the safe and effective weight management methods during pregnancy. Late ANC booking was associated with excessive GWG (P=0.002).

**Conclusion:**

maternal obesity and excessive GWG is highly prevalent among ANC clients in the Fako Division, with excessive GWG being associated with late ANC booking. Hence, there is a need to design community-based interventions that could increase rates of early booking visits and consequently increase its benefits.

## Introduction

Obesity is a significant non-communicable disease risk factor [1], which describes excessive body fat accumulation that adversely affects health [2]. Based on Body Mass Index (BMI) obesity is BMI ≥30 Kg/m^2^ [3]. Gestational weight gain (GWG) is the weight gained during pregnancy (from conception to the birth of an infant) [4]. There is a recommended range of GWG based on maternal pre-pregnancy BMI [5], and high GWG (>25kg) is associated with risks of being overweight and obese after pregnancy [6].

Maternal obesity is a public health problem [7]. Its increasing incidence globally is associated with short- and long-term complications in mothers and child [8]. Pregnancy is an obesity trigger [9], with the prevalence of obesity in pregnancy at 30% and 40% for women with adequate and excessive GWG respectively [7]. Amongst Cameroonian women, the prevalence of obesity is 24.1% [10] and prevalence of excessive GWG at 24% to 34% [11,12]. Black women are more likely to be overweight pre-pregnancy and gain gestational weight [13]. Maternal obesity is associated with gestational diabetes, hypertension in pregnancy, venous thromboembolism, caesarean delivery, and stillbirth. Also, it is a risk factor for spontaneous abortion, obstructed labour and increased perinatal risks [14].

There are independent associations between maternal obesity, macrosomia and childhood obesity [15]. These obese children become obese adults, hence pregnancy is a critical window of opportunity for prevention of obesity now and in the future [15]. Most pregnant women have no concerns about maternal obesity due to ignorance about their BMI, GWG target limits, and appropriate weight management strategies [16]. Dieting, physical activity and behavioural counselling positively affect weight loss and obstetric outcomes [17]. In Cameroon, there is paucity of data on the prevalence of obesity in pregnancy and the knowledge of pregnant women on obesity, its complications, and weight management techniques during pregnancy. Hence, this study aims to determine the prevalence of obesity in pregnancy and excessive GWG and determine the factors associated with obesity in pregnancy, assess the level of knowledge on the complications of obesity in pregnancy and safe weight management among pregnant women attending ANC in the Fako Division, Cameroon.

## Methods

**Study design and setting:** we conducted a hospital-based cross-sectional study from 28^th^ January 2020 to 29^th^ May 2020, at the Limbe District Hospital (LDH) and Buea Road Integrated Health Centre (BRIHC). These primary level health care facilities are located in the Limbe health district and Buea health district, respectively. The facilities are in urban areas within the Fako Division, which contains 6 healthcare facilities. These facilities were selected due to the high number of ANC consultations within their respective health districts. Women attending antenatal care in these facilities tend to come from Buea, Limbe and Tiko which are the urban and semi-urban areas within the Fako Division.

**Study population and sampling:** the target population were women attending ANC in the LDH and BRIHC. We included into study women with singleton pregnancy confirmed by ultrasound with pre-pregnancy maternal weight measurements that attended ANC and signed an informed consent/assent form at the LDH and BRIHC. We excluded women with multiple gestations, those who did not consent, and those with no record of their pre-pregnancy weight. The sample size estimation was with Cochran's formula [18] with the assumptions of 95% confidence interval, 7.4% prevalence of maternal obesity from a previous study in Nigeria [19] and a 5% margin of error. The minimum sample size was calculated at 106 participants but considering a non-response rate of 10%, and we had a target of 117 participants. An estimated facility sample size of 61 and 57 women was calculated for the LDH and BRIHC respectively using the average influx of patients in each facility per month.

**Ethical approval:** for this study, it was obtained from the Institutional Review Board of the Faculty of Health Sciences, University of Buea with reference number 2020/1049-01/UB/SG/IRB/FHS. Participants were required to sign a consent form or an assent form prior to inclusion.

**Data collection:** we retrieved the pre-pregnancy and previous weights from the patient's case note so as to prevent any recall or information bias as some of the participants could not remember their pre-pregnancy weight. We included pre-pregnancy weight of up to 6 months before pregnancy. Participants’ weight and height were measured using instruments available at the facility. The women then filled a pretested questionnaire which was adapted from questionnaire used from a similar study carried out in Australia [16] (Annex 1). Information collected included socio-demographic characteristics, anthropometric data, history, questions assessing the women's knowledge of their BMI, GWG, complications of maternal obesity and safe weight management techniques during pregnancy. After collecting data, participants were informed about their BMI, GWG and educated on healthy weight gain and nutrition during pregnancy.

**Definitions:** dependent variables were obesity in pregnancy and excessive GWG. Independent variables included age, level of education, occupation, parity, residence, alcohol, and smoking. Continuous variables like age, BMI, parity, gravidity and booking visit timing were categorised during data analysis. They were categorised as age <20, 20-29 and 30-39, BMI as Underweight, normal weight, overweight and obese, Parity as nulliparous, primiparous, multiparous and grand multiparous, Gravidity primigravid, multigravida and grand multigravida, and Booking visit timing as early and late.

Late Booking visit was defined as first official ANC appointment on or after the 14^th^ week of gestation.

Obesity in pregnancy was defined using the participants BMI. Women with BMI ≥30 were classified as obese, those with BMI of 25.0-29.9 were overweight, those with BMI 18.5-24.9 as normal weight and those with BMI ≤ 18.5 as underweight. This definition was used in several studies [20-23].

GWG was defined based on the IOM recommendations. Based on the pre-pregnancy BMI (which was obtained using the pre-pregnancy weight and the height), adequate GWG was defined as 12.5-18kg for underweight, 11.5-16kg for normal weight, 7-11.5kg for overweight, and 5-9kg for obese women. Depending on the participants trimester of pregnancy GWG was obtained using a pregnancy weight gain calculator based on IOM recommendations [24].

For the knowledge of complications, there were 18 checkbox questions with a total of 18 points. The correct box checked was considered 1 point, while the wrong box checked was 0 points. We categorized the knowledge level score as good (≥ 7 points), moderate (4 - 6 points), poor (1-4 points) and no knowledge (0 points).

For knowledge on the safe weight management techniques, there were with 15 questions with a total of 15 points. The answer yes (1), no (0), do not know (0) points, respectively. This was categorised as good (11-15 points), moderate (6 - 10 points) and poor (0-5 points).

**Statistical analysis:** data were entered into an excel spreadsheet, exported to, and analysed with IBM SPSS version 26.0. Frequencies and percentages were computed for categorical variables. Mean (or median) and standard deviation from continuous variables. Logistic regression model was used to analyse associations between maternal obesity, and different determinants in order to obtain the factors associated with maternal obesity. Chi-square tests was used to find associations between categorical variables. Significant P-value was set at <0.05 at a 95% CI. After the univariate analysis, variables with P-value <0.2 were included in the multivariate analysis.

## Results

A total of 395 pregnant women were approached to participate in the study, 78 participants did not have a medical record of pre-pregnancy weight and hence were excluded, giving a response rate of 80.3%.

**Socio-demographic and obstetric characteristics:** participants ranged from 17 to 39 years with a mean age of 27.7 ± 5.1 years; 58.4% were between 20 to 29 years of age. Of the 317 participants, 57.4% (182) attended secondary school, and 74.8% (237) were married. Thirty-six per cent were unemployed. The mean gestational age at the time of the study was 28.82 ± 7.75 weeks (Table 1).

**Table 1:** socio-demographic and obstetric characteristics of participants

Variable	Frequency (N=317)	Percentage
**Age group (years)**		
< 20 years	16	5.0
20-29 years	185	58.4
30-39 years	116	36.6
**Marital status**		
Single	80	25.2
Married	237	74.8
**Education**		
Primary	29	9.1
Secondary	182	57.4
Tertiary	104	32.8
Never	2	0.6
**Occupation**		
Civil service	18	5.7
Private sector	50	15.8
Self-employed	133	42
Unemployed	116	36.6
**Residence**		
Urban	246	77.6
Rural	71	22.4
**Parity**		
Nulliparous	105	33.1
Primiparous	102	32.2
Multiparous	104	32.8
Grand multiparous	06	1.9
**Booking visit**		
Early	59	18.6
Late	258	81.4

**Prevalence of maternal obesity and excessive GWG:** seventy three (23%) participants were obese pre-pregnancy, 106 (33.4%) at their first ANC visit, and 134 (42.3%) at the time they were recruited into the study. Of the 134 obese pregnant women, 90 (67.2%) were in class I, 34 (25.4%) in class II and 10 (7.4%) were in class III. Also, 132 (41.6%) participants had gain excessive gestational weight.

**Factors associated with maternal obesity:** on a univariate analysis, obesity in pregnancy was associated with participant's age, marital status, occupation, alcohol consumption, gravidity and parity. On multivariate analysis, alcohol consumption (aOR: 2.11, 95% CI 1.19-3.71; p; = 0.01) was associated with obesity in pregnancy. Also, women aged 20-29 years (aOR: 0.297, 95% CI 0.16-0.56; p<0.001) and those <20 years (aOR: 0.064, 95% CI 0.007-0.57; p= 0.014) were less likely have maternal obesity compared to those aged 30-39 years (aOR: 1) (Table 2, Table 3).

**Table 2:** relationship between participants actual BMI and perceived BMI

	Perceived weight category			
Actual BMI	Normal weight	Obese	Overweight	Underweight
Normal	47 (14.8%)	0 (0.0%)	5(1.6%)	7(2.2%)
Obese	71 (22.4%)	9 (2.8%)	48 (15.1%)	6(1.9%)
Overweight	89(28.1%)	2(0.6%)	24 (7.6%)	9 (2.8%)

BMI: Body Mass Index

**Table 3:** univariate and multivariate analysis

Predictors	Levels	P-value	OR (95%CI)	P-value	AOR (95% CI)
**Age group**	< 20	0.001	0.034 (0.004-0.265)	0.014	0.064 (0.007-0.570)
	20 - 29	<0.001	0.220 (0.134-0.361)	<0.001	0.297 (0.160-0.550)
	30 - 39	<0.001		.	1
**Gravidity**	1	0.002	0.341 (0.170-0.684)	0.159	6.242 (0.488ï¿½7-9.853)
	2	0.032	0.475 (0.24-0.939)	0.215	2.434 (0.596-9.935)
	3	0.687	1.159 (0.566-2.370)	0.116	2.40 (0.805-7.157)
	4 +	<0.001		.	1
**Parity**	0	<0.001	0.225 (0.10-0.51)	0.111	0.120 (0.009-1.624)
	1	0.017	0.381 (0.173-0.842)	0.183	0.359 (0.079-1.621)
	2	0.583	0.794 (0.348-1.811)	0.385	0.573 (0.163-2.016)
	3+	<0.001			1
**Alcohol consumption**	Yes	<0.001	2.22 (1.321-3.72)	0.013	2.081 (1.167-3.711)
	No				1
**Education**	Primary	0.150	1.815 (0.806-4.089)	0.997	0.998 (0.359-2.775)
	Secondary	0.481	0.839 (0.514-1.368)	0.980	0.992 (0.522-1.885)
	Tertiary				1
**Occ**	Civil service	0.061	2.63 (0.96-7.21)	0.939	1.046 (0.329-3.326)
	Private sector	0.001	3.34 (1.67-6.67)	0.108	1.885 (0.871-4.082)
	Self-employed	0.001	2.51 (1.48-4.27)	0.162	1.558 (0.837-2.897)
	Unemployed				1
**ANC hospital**	BRIHC	0.104	1.45 (0.93-2.27)	0.325	1.304 (0.77-2.21)
	DHL				1
**Marital status**	1	0.014	0.51 (0.295-0.874)	0.286	0.698 (0.361-1.351)
	2				
**Number of ANC sessions attended**	1	0.961	1.02 (0.499-2.074)		
	2	0.940	1.03 (0.536-1.962)		
	3	0.464	o.76 (0.359-1.596)		
	4+	0.876			

**Association between maternal age, timing of booking visit and excessive GWG**

We also found a statistically significant association between timing of booking visit and excessive GWG (P=0.002, chi-square = 11.27) with women who initiated their booking visit late more likely to gain excessive gestational weight. Participants age groups were also significantly associated (P= 0.003, Chi-square = 4.664) with excessive GWG, with women aged 30 - 39 years more likely to gain excessive weight.

**Knowledge on BMI and GWG:** about half of the respondents (52.7%) did not know their recommended GWG: 44 (13.9%) of the women correctly estimated their recommended GWG, 74 (23.3%) underestimated their recommended GWG and 32(10.1%) overestimated their recommended GWG. 80 participants (25.2%) correctly identified their BMI category (Table 2). and we found a statistically significant association (p<0.001, chi-square = 30.44) between perceived weight category and actual BMI category.

**Knowledge on complications of obesity and excessive GWG:** two hundred and thirty two participants (73.1%) agreed that there are maternal complications of obesity in pregnancy while 81 participants (25.6%) said there are no maternal complications of obesity in pregnancy. 4 participants (1.3%) did not know if there were any maternal complications. Out of 232 women who agreed that there are maternal complications, 64 (14.2%) did not know any of the specific complications. 173 (54.6%) participants agreed that there are foetal complications of maternal obesity while 138 (43.5%) women said there are no foetal complications of maternal obesity. 6 of them (1.9%) did not know if there were foetal complications. Of the 137 participants who agreed that there are foetal complications, 73 (16.2%) did not know any specific foetal complication. Combining the knowledge on maternal and foetal complications, 41% (n=130) had no knowledge, 46.1% (n=147) had poor knowledge, 8.8% (n=27) had moderate knowledge and 4.1% (n=13) had good knowledge.

**Knowledge on safe and effective methods of weight management in pregnancy:** the overall knowledge level on the safe and effective weight management methods in pregnancy was moderate 245 (77.3%), with only 61 (19.2%) having good knowledge. Study participants had many incorrect beliefs about safe weight management in pregnancy. Although 84.5% though that they do not need to avoid exercise because they are pregnant, only 62.8% agreed that they needed to exercise 3 or more times each week. Also, 181 (57.1%) of the participants thought they should eat a double portion of food because they are pregnant (Table 4).

**Table 4:** pregnant women's specific beliefs about safe and effective management of weight gain in pregnancy

	Expert opinion	Number(%) of participants answering correctly
**Dietary behaviour**		
Skip meals	No	280 (88.3)
Eat a double portion of food	No	115 (36.3)
Remove fat from meat	Yes	208 (65.6)
Stop eating after eight pm at night	No	85 (26.8)
**Dietary approaches**		
Choose low-fat milk and dairy products	Yes	253(79.8)
Eat fewer cakes and chocolates	Yes	278 (87.7)
Drink less soft drinks	Yes	271 (85.5)
Drink more fruit juice	No	23 (7.3)
Eat plenty of fruits and vegetables	Yes	312 (98.4)
Eat less fried foods	Yes	234 (73.8)
Eat low carbohydrate diet	No	110 (34.7)
Eat less oil	Yes	269 (84.9)
**Exercise**		
Avoid exercise	No	268 (84.5)
Exercise 3 or more times each week	Yes	199 (62.8)

## Discussion

The aim of our study was to determine the prevalence of obesity in pregnancy and excessive GWG, determine the factors associated with obesity in pregnancy, assess the level of knowledge on its complications and safe weight management in pregnancy. The prevalence of obesity and excessive GWG was 42.3% and 41.6% respectively. Older women and those who consumed alcohol were more likely to be obese. Most women had poor knowledge on complications of obesity and moderate knowledge on safe weight management techniques.

The prevalence of obesity in pregnancy was 42.3%. This was lower than 50.7% reported from a study in Nigeria [20] which could be because the Nigerian study used only third-trimester measurements whereas we included women in all trimesters. A study in Douala, Cameroon, reported a lower prevalence of excessive GWG and a higher prevalence of insufficient GWG [25] contrary to our findings which could be because most of the women in our study were unemployed or had an unstable source of income, making them more likely to consume food with high caloric value [26]. Also, increase in maternal awareness of insufficient GWG, could have led to these women having a greater preoccupation with too little food intake and weight gain rather than too much. An increase in sedentary lifestyle over the years within the Fako Division with more taxis, reduced sidewalks and no public parks could have contributed to this higher prevalence of maternal obesity and excessive GWG.

We found alcohol consumption in pregnancy to be associated with gestational obesity. Many obese pregnant women had pre-gestational obesity, and alcohol is reportedly associated with pre-gestational obesity [27]. Other studies have implicated alcohol consumption during pregnancy to affect excessive GWG [28]. Increasing maternal age was associated with maternal obesity is consistent with other studies [21,29,30].

Our findings on the women's knowledge about the maternal and foetal complications of obesity in pregnancy were similar to those of a study in Nigeria [31]. We also found that the poor knowledge of the specific consequences of maternal obesity on mother and child conform with results from Nigeria and Australia [16,31]. Most women had better knowledge of the weight management techniques compared to complications of maternal obesity and excessive GWG. Although knowledge on weight management is essential, knowing the adverse effects of being obese and gaining more weight than is recommended might increase their motivation as they would not want to be victims of the adverse effects. A third of our participants believed that avoiding energy giving foods was a safe way to manage obesity in pregnancy and given our setting in Cameroon, where there is still a significant percentage of underweight women [10], this belief may result in women avoiding foods that provide much-needed energy. Hence, interventions should educate women on the complications of being obese and gaining excessive weight, and the appropriate nutritional intake with emphasis on eating a balanced diet rather than avoiding food types [32].

More than a third of our participants believed that exercising three or more times per week was too much for pregnant women which could be due to the environment's conduciveness to exercise, cultural beliefs, inadequate knowledge of the types of exercises appropriate for pregnant women or the benefits of exercising during pregnancy [33]. The current trends recommend that pregnant women eat a balanced diet, limit portion sizes, and avoid losing weight during pregnancy as this can be harmful [32]. The American College of Obstetrics and Gynaecology recommends that pregnant women partake in moderate exercises for 30 minutes [34]. However, there are no specific recommendations from the Society of Gynaecologists and Obstetricians of Cameroon (SOGOC) addressing maternal obesity and excessive GWG among Cameroonian women.

Bridging the knowledge gap on maternal obesity, GWG, consequences, and management strategies among pregnant women is essential to improving perinatal outcomes, especially for those who are overweight or obese pre-pregnancy [16]. Many pregnant women's ideas of an appropriate diet in pregnancy may diverge from expert opinion. A study in South-western Nigeria reported that approximately one-quarter of women in the study received counselling on weight gain from family and friends [35]. So health care providers should not assume that pregnant women are using safe strategies to avoid excessive GWG.

Women who booked late for ANC were more likely to gain excessive weight than women who booked early because early ANC booking leads to more ANC sessions attended during that pregnancy with longer follow-up period, making them more knowledgeable about the complications of excessive weight gain and the safe practices to prevent excessive GWG and ensure optimal nutrition. A similar carried out in Nigeria showed that women who booked early were more likely to have a better knowledge of maternal and child complications and better manage their weight during pregnancy to prevent excessive GWG [31]. Women should, therefore, be encouraged to initiate ANC early.

The lack of knowledge of personal BMI, GWG target limits, and appropriate weight management strategies limits the ability of women to address these issues successfully during pregnancy. Pregnant women and those of reproductive age should not only be encouraged to start their antenatal visits early but also pay attention to and comply to the advice provided during the health education talks. During ANC sessions, the content and teaching of nutrition programmes should include using teaching aids like posters of different food items and practical food demonstrations.

Our study is among the first few studies to explore pregnant women's knowledge on maternal obesity and Excessive GWG using a wide range of variables that may represent the situation that occurs in other sub-Saharan countries. Also, given that the target population was pregnant women, the findings from this study shows that interventions delivered to pregnant women have the potential to improve mother, child and perinatal outcomes. Despite these strengths, this study was subject to recall and reporting bias and to reduce these, we used the pre-pregnancy weights which had been recorded in the participants case notes. We also pretested the questionnaire for design flaws as part of the validation instrument.

## Conclusion

We had a 42.3% prevalence of maternal obesity and a 41.6% prevalence of excessive GWG, with older women and those who consume alcohol during pregnancy being more likely to be obese. Most participants had poor knowledge on the maternal and foetal complications of maternal obesity and had moderate knowledge level on the safe weight management techniques during pregnancy. Many women started their ANC late, and they were more prone to excessive GWG. Women should therefore be encouraged to initiate their ANC early and comply with the advice provided during these sessions. It is also crucial to use ANC visits as an opportunity to educate and improve awareness on the safe weight management techniques and debunk myths around maternal obesity and GWG.

### What is known about this topic

- *Increasing prevalence of maternal obesity and excessive GWG in developed countries;*
- *Safe and effective weight management techniques can be used to prevent excessive GWG and maternal obesity*.

### What this study adds

- *There is a significant prevalence of excessive GWG and maternal obesity among pregnant women in the Fako Division, Cameroon;*
- *Older women and those who consumed alcohol during pregnancy are at higher risk maternal obesity;*
- *Pregnant Women in the Fako Division have poor knowledge on the complications of maternal obesity; many pregnant women initiate ANC late, and these women are likelier to gain excessive weight during pregnancy*.

## Annexe

**Annex 1**: legend pretested questionnaire adapted from a similar study (PDF - 471 KB)
